# Silica Nanoparticles Promote α-Synuclein Aggregation and Parkinson’s Disease Pathology

**DOI:** 10.3389/fnins.2021.807988

**Published:** 2022-01-13

**Authors:** Xin Yuan, Yingxu Yang, Danhao Xia, Lanxia Meng, Mingyang He, Chaoyang Liu, Zhentao Zhang

**Affiliations:** ^1^Department of Neurology, Renmin Hospital of Wuhan University, Wuhan, China; ^2^Hubei Provincial Institute for Food Supervision and Test, Wuhan, China; ^3^Research Center for Environment and Health, Zhongnan University of Economics and Law, Wuhan, China

**Keywords:** silica nanoparticle, neurodegeneration, Parkinson’s disease, α-synuclein, inhalation exposure

## Abstract

Silica nanoparticles (SiO_2_ NPs) are increasingly investigated for their potential in drug delivery systems. However, the neurotoxicity of SiO_2_ NPs remains to be fully clarified. Previously SiO_2_ NPs have been reported to be detected in the central nervous system, especially in the dopaminergic neurons which are deeply involved in Parkinson’s disease (PD). In this article, we characterized the effects of SiO_2_ NPs on inducing PD-like pathology both *in vitro* and *in vivo*. Results showed that SiO_2_ NPs promote more severe hyperphosphorylation and aggregation of α-synuclein, mitochondria impairment, oxidative stress, autophagy dysfunction, and neuronal apoptosis in the α-Syn A53T transgenic mice intranasally administrated with SiO_2_ NPs compared with the control group. Our findings provide new evidence supporting that SiO_2_ NPs exposure might have a strong capability of promoting the initiation and development of PD.

## Introduction

Silica nanoparticles (SiO_2_ NPs) are defined as nano-sized (1-100 nm) silicon dioxide. They hold a tremendous surface/volume ratio and manifest remarkable surface reactivity compared to the bulk forms with larger diameters (Murugadoss et al., 2017). SiO_2_ NPs are recently investigated in drug delivery, genetic therapy, molecular imaging, and the potential of antibiosis due to their unique physicochemical features (Chen et al., 2018). Currently, SiO_2_ NPs exposure contributes a rapidly growing part in air pollution and rises a threat to human health (Mohammadinejad et al., 2019).

A lot of approaches are responsible for SiO_2_ NPs entering the internal environment such as respiratory tract inhalation, digestive tract intake, skin contact, and intratracheal instillation (Guo et al., 2021), and they significantly deteriorate multiple organs and systems (Yamashita et al., 2011; Yoshida et al., 2011; Nabeshi et al., 2012; Inoue et al., 2021). Both *in vitro* and *in vivo* studies have proved that SiO_2_ NPs significantly induce pathological alterations in the brain (You et al., 2018; Wei et al., 2020). They demonstrate a strong capability of invading the central nervous system by intranasal instillation and preferentially deposit in the striatum (Wu et al., 2011). SiO_2_ NPs even manifest neurotoxicity via the gut-brain axis by oral administration (Diao et al., 2021). They also promote the deposition of intracellular amyloid-β (Aβ) and hyperphosphorylation of tau in neuro2a neuroblastoma cells. All these results raised the possibility that nanoparticle counts for the onset and development of Alzheimer’s disease (AD) (Yang et al., 2014; Huang et al., 2015).

Parkinson’s disease is characterized by the loss of dopaminergic neurons in the substantia nigra pars compacta (SNpc) and intracellular α-synuclein (α-Syn) aggregation (Hijaz and Volpicelli-Daley, 2020). Although it is one of the most common neurodegenerative disorders, the pathogenesis of PD remains to be elusive. Both environmental and genetic factors contribute to the initiation of typical PD-like pathological degeneration (Dunn et al., 2019). Mitochondrial dysfunction, oxidative stress, autophagy, and misfolded α-Syn aggregation have been implicated in PD pathology (Jankovic and Tan, 2020). SiO_2_ NPs have been reported to induce behavioral impairment in zebrafish (Li et al., 2014, 2020). However, the effects of SiO_2_ NPs exposure on Parkinson’s disease (PD) pathology remains unknown.

In this article, we tested the effects of SiO_2_ NPs on triggering α-Syn deposition and dopaminergic neuron death. It showed that SiO_2_ NPs promote α-Syn aggregation both *in vitro* and *in vivo* and they induce mitochondria impairment and autophagy dysfunction in cellular models. Intranasal instillation of SiO_2_ NPs to transgenic mice expressing A53T human α-Syn enhanced the α-synucleinopathy and dopaminergic neuronal degeneration. Therefore, SiO_2_ NPs exposure significantly promotes PD pathology.

## Materials and Methods

### Reagents

The following antibodies and reagents were used: pα-Syn (Ser129, Biolegend, 825701), pα-Syn (Ser129, Cell Signaling Technology, 23706s), MAP2 (Thermo Fisher Scientific, SF254293), COX IV (Abcam, ab16056), ATG5 (Proteintech, 10181-2-AP), Beclin1 (Proteintech, 11306-1-AP), LC3 (Cell Signaling Technology, 12741), Bcl2 (Cell Signaling Technology, 3498S), Bax (Proteintech, 50599-2-Ig), GAPDH (Proteintech, 60004-1-Ig), TH (Sigma-Aldrich, AB152), Ubiquitin (Santa Cruz Biotechnology, sc-8017), Iba-1 (Wako, 019-19741), Alexa Fluor 594-conjugated goat anti-mouse IgG (Invitrogen, A-11005), Alexa Fluor 488-conjugated goat anti-rabbit IgG (Invitrogen, A-11012), DAPI (Biofroxx, EZ3412B205), HRP-conjugated anti-mouse IgG (BIO-RAD, 170-6516), HRP-conjugated anti-rabbit IgG (BIO-RAD, 170-6515), Complex I Enzyme Activity Microplate Assay Kit (Abcam, ab109721), and Reactive Oxygen Species Assay Kit (Nanjing Jiancheng Bioengineering Institute, E004-1-1).

### Purification and Aggregation of Recombinant Human α-Synuclein

Full-length α-Syn was purified as previously described (Volpicelli-Daley et al., 2014). His-tagged α-Syn were expressed in Escherichia coli BL21 (DE3). Bacterial pellets were resuspended in 100 mL osmotic shock buffer (30 mM Tris-HCl, 40% sucrose, 2mM ethylenediaminetetraacetic acid disodium, pH 7.2) and incubated for 10 min at room temperature, and then centrifuged at 12,000 rpm for 20 min. The pellets were resuspended quickly in 90 mL cold water with 37.5 μL saturated MgCl_2_, and centrifuged at 12,000 rpm for 20 min. The supernatants were applied onto a Ni-chelating affinity chromatography and eluted at 125 mM imidazole. α-Syn preformed fibrils (PFFs) were prepared by incubating protein at 37°C with constant shaking at 1,000 rpm for 7 days. Protein fibrillization was confirmed using the thioflavin T fluorescence assay. α-Syn preformed PFFs were sonicated with 60 pulses at 10% power (total of 30 s, 0.5 s on, and 0.5 s off) before use.

### Characterization of Silica Nanoparticles

Silica nanoparticles were purchased from Sigma-Aldrich (St. Louis, MO, United States). The average size and morphology were confirmed by transmission electron microscopy (TEM, HT7800/HT7700, Hitachi, Japan). Simply, 3 μL of SiO_2_ NPs (2 μg/μL) were adsorbed onto a carbon-coated 200-mesh gird for 1 min, washed with Milli-Q water (3 × 10 μL), allowed to dry at room temperature, and then negatively stained with 2% uranyl acetate. Finally, the gird was viewed at 80 kV under the TEM. The hydrodynamic diameter of SiO_2_ NPs in distilled water and DMEM/F12 medium with 10% fetal bovine serum (FBS) were measured using dynamic light scattering (DLS, Nano-S90, Malvern Instruments, United Kingdom). SiO_2_ NPs were suspended in PBS to a concentration of 10 μg/μL for cell experiments, and 5 μg/μL for animal experiments. Suspensions were sonicated for 30 min to be fully homogenized before use.

### Cell Culture and Silica Nanoparticles Treatment

SH-SY5Y cells and HEK293 cells stably expressing GFP-α-Syn (termed as HEK293-α-Syn cells) were cultured in DMEM/F12 medium with 10% fetal bovine serum and 100 μg/mL Ampicillin-Streptomycin. Cells were cultured at 37°C in an atmosphere containing 5% CO_2_. For α-Syn seeding experiment, SH-SY5Y cells and HEK293-α-Syn cells were pretreated with SiO_2_ NPs for 24 h, and then 10 μg α-Syn PFFs were added into the medium. For cytotoxicity experiments, SH-SY5Y cells were treated with SiO_2_ NPs for 48 h. SiO_2_ NPs were added in the culture medium to final concentrations of 100 and 200 μg/mL.

### Primary Neuron Cultures

Primary mouse cortical neurons dissected from A53T transgenic mice embryos were cultured as previously described (Zhang et al., 2021). On the seventh day, neurons were treated with α-Syn PFFs and SiO_2_ NPs and cultured for an additional 7 days. On the 14th day, cells were fixed in 4% formaldehyde, permeabilized, and then subjected to immunofluorescence analysis. The Olympus IX73 microscope mounted with a DP80 Olympus digital camera was used for image capture.

### Complex I Enzyme Activity Analysis

Mitochondria were isolated using the Cell Mitochondria Isolation Kit (Beyotime, Shanghai, China) according to the product manual. Cells were harvested and washed with ice-cold PBS, and then ice-bathed in 2 mL mitochondria isolation solution for 15 min before being homogenized 30 times using a glass homogenizer. The solutions were centrifuged at 1,000 g for 10 min and the pellets were removed. The supernatants were remained and centrifuged at 11,000 g for 10 min at 4°C. The pellets containing mitochondria were suspended in 150 μL mitochondrial storage fluid and subjected to the Complex I activity assay immediately. Complex I Enzyme Activity Microplate Assay Kit was used for assessing the bioactivity of mitochondrial Complex I. Briefly, 200 μL of mitochondria were added to the pre-coated microplate and incubated for 3 h at room temperature. After washing three times, a 200 μL working buffer was applied. The optical density (OD) at 450 nm was monitored for 30 min.

### Measurement of Reactive Oxidative Species

ROS in SH-SY5Y cells was measured using the Reactive Oxygen Species Assay Kit following the product instruction. After 48-h SiO_2_ NPs treatment, cells were washed with PBS and incubated with 1 mL DCFH-DA solution (1:1000, dissolved in culture medium) for 30 min at 37°C. After being washed with PBS, cells were imaged with a fluorescence microscope. The integrated fluorescence density was quantified using ImageJ software (version 2.1.0/1.53c).

### Propidium Iodide and Hoechst 33258 Double Staining

Apoptosis of SH-SY5Y cells was quantified by double immunofluorescence labeling with propidium iodide (PI, Beyotime, Shanghai, China) and Hoechst 33258 (Beyotime, Shanghai, China). After treatment with SiO_2_ NPs for 48 h, cells were washed with PBS and incubated with Hoechst33258 (10 μg/mL) and PI (10 μg/mL) solution for 30 min at 4°C. Cells were washed three times with PBS before fluorescence microscopy. Bright red-stained nuclei were considered apoptotic. Apoptosis ratio% = Apoptotic cells (n)/Total number of cells (n).

### Animals

Male transgenic mice expressing A53T human α-Syn (α-Syn A53T Tg mice) were housed in the Animal Experiment Center of Renmin Hospital of Wuhan University. Animal handling was in accordance with the Experimental Animal Management Criterion and approved by the Ethics Committee of the Renmin Hospital of Wuhan University (IACUC Issue No. WDRM 20210319). Mice (23–26 g) at the age of 3 months were randomly divided into two groups (SiO_2_ NPs and PBS groups). Since the natural exposure dose of humans to SiO_2_ NPs is approximately 2.7–15.53 mg/kg by intranasal instillation (You et al., 2018), 5 μg/μL SiO_2_ NPs were delivered to mice by intranasal instillation to a total volume of 15 μL PBS or SiO_2_ NPs solution every 2 days for 3 months.

### Western Blots

The cells were lysed in NP-40 buffer with protease inhibitor and centrifuged at 15,000 rpm at 4°C for 15 min. Supernatants were quantified by BCA assay and subjected to Western blot analysis. After SDS-PAGE, proteins were transferred onto a nitrocellulose membrane, blocked with 5% milk, and incubated with primary antibodies at 4°C overnight. HRP-conjugated secondary antibodies were accordingly applied for incubation for 1 h at room temperature. After washing 3 times in TBST, signals were developed with enhanced chemiluminescent.

### Immunostaining

For immunocytochemistry, primary neurons were fixed in 4% PFA and 0.1% TritonX-100 for 20 min and then washed with PBS. After blocking for 30 min in 5% BSA, the neurons were incubated with primary antibodies overnight at 4°C. After washing 3 times in PBS, secondary antibodies were applied for incubating 2 h at room temperature, and then the cells were washed with PBS 3 times followed by DAPI labeling. The glass cover carrying cells were mounted using glycerol and examined with fluorescence microscopy. For immunohistochemistry and immunofluorescence, paraffin-embedded brain sections were deparaffinized, hydrated, and incubated in antigen retrieval buffer (0.1 M sodium citrate, pH 6.0) at 94°C for 20 min. After being blocked with 5% BSA for 30 min, the sections were incubated with primary antibodies at 4°C overnight. High-Efficiency IHC Detection Kit (Absin, abs957) was used for signal development.

### Statistical Analysis

All data were presented as Mean ± SEM and analyzed by GraphPad Prism (version 8.2.0). The student’s *t*-test was used for analyzing the differences between two or more groups. Ordinary one-way ANOVA followed by Tukey’s multiple comparisons were used for analyzing three or more groups. Mann-Whitney *U* test was applied to assess the ratio differences between the two groups. For data consist two independent variables, two-way ANOVA following Bonferroni’s multiple comparisons was performed. A *P* less than 0.05 was considered statistically significant.

## Results

### Characterization of Silica Nanoparticles

**FIGURE 1:**
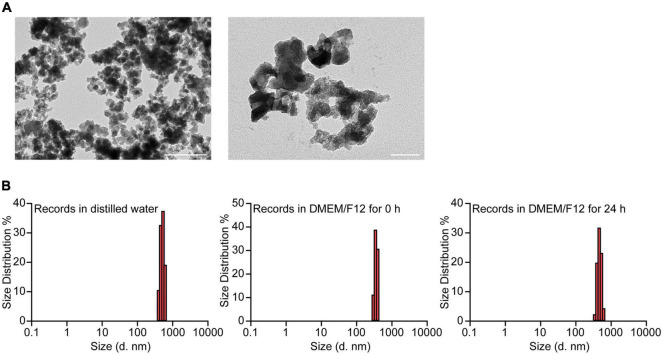
Characterization of SiO_2_ NPs. **(A)** TEM analysis of SiO_2_ NPs. Scale bar = 400 nm, 100 nm. **(B)** Hydrodynamic sizes of SiO_2_ NPs in distilled water, DMEM/F12, and DMEM/F12 for 24 h.

The morphology of the SiO_2_ NPs was characterized by TEM (Figure 1A). Since the SiO_2_ NPs used in this study were suspended in DMEM/F12 medium with 10% FBS, we evaluated the hydrodynamic properties by DLS (Figure 1B). The mean hydrodynamic diameters of the SiO_2_ NPs in distilled water, DMEM/F12 with 10% FBS, and DMEM/F12 with 10% FBS for 24 h were 509.3, 356.0, 469.0 nm, respectively.

### Silica Nanoparticles Promote α-Syn Aggregation *in vitro*

**FIGURE 2:**
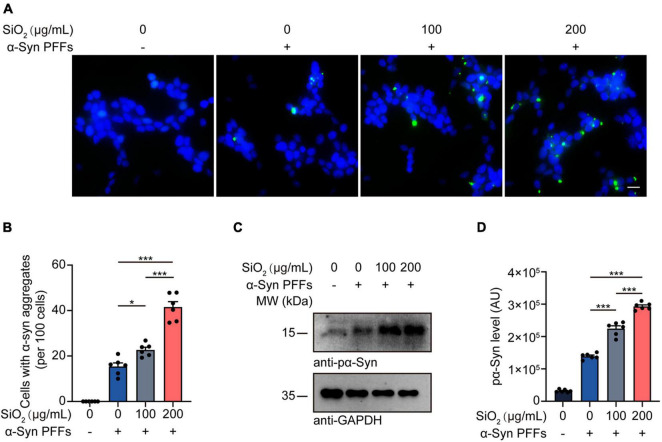
SiO_2_ NPs promote α-Syn aggregation in HEK293-α-Syn cells. **(A,B)** Fluorescence analysis showing α-Syn aggregates in HEK293-α-Syn cells treated with SiO_2_ NPs and α-Syn PFFs. Scale bar = 20 μm (mean ± SEM; *n* = 6 per group; *P < 0.05, ***P < 0.001, one-way ANOVA). **(C,D)** Western blot analysis of pα-Syn levels in HEK293-α-Syn cells treated with different concentrations of SiO_2_ NPs and α-Syn PFFs (mean ± SEM; *n* = 6 per group; ***P < 0.001, one-way ANOVA).

**FIGURE 3:**
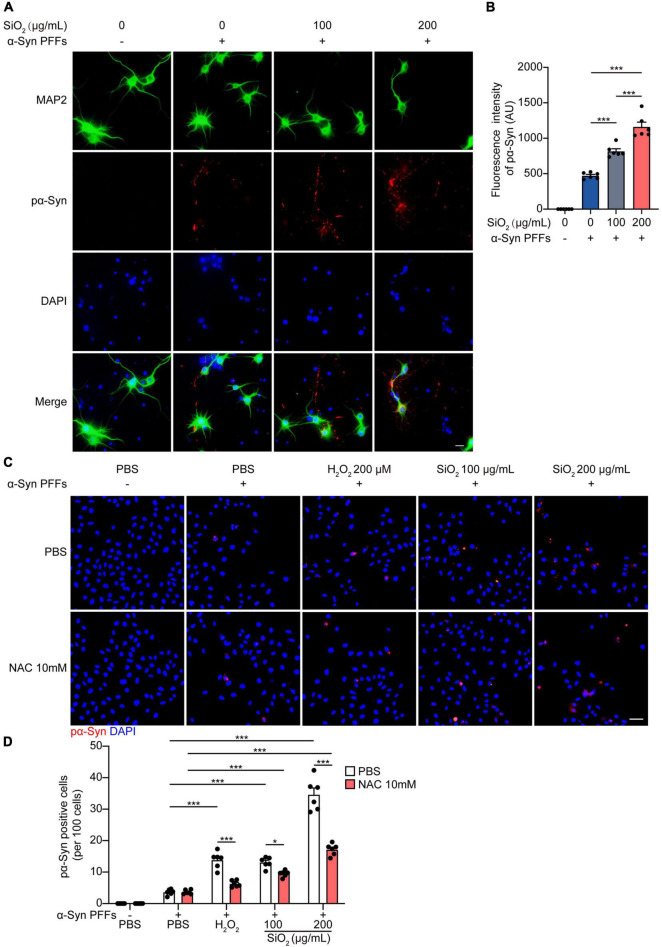
SiO_2_ NPs promote α-Syn aggregation in neurons. **(A,B)** Immunofluorescence staining of pα-Syn and MAP2 in primary neurons from α-Syn A53T Tg mice. Scale bar = 20 μm (mean ± SEM; *n* = 6 per group; ***P < 0.001, one-way ANOVA). **(C,D)** Immunofluorescence staining of pα-Syn in SH-SY5Y cells, scale bar = 50 μm (mean ± SEM; *n* = 6 per group; *P < 0.05, ***P < 0.001, two-way ANOVA).

To investigate the effect of SiO_2_ NPs on the aggregation dynamics of α-Syn, we used the HEK293-α-Syn cell line as a phenotyping panel. As reported previously, α-Syn inclusions would not spontaneously emerge in the cytoplasm of the HEK293-α-Syn cells overexpressing GFP-tagged α-Syn unless we artificially introducing exogenous α-Syn PFFs (Sanders et al., 2014). The cells were treated with SiO_2_ NPs for 24 h before the α-Syn PFFs were added into the medium with lipo2000. We found that pre-treatment with SiO_2_ NPs promoted the aggregation of α-Syn in a dose-dependent manner, but the crystal SiO_2_ does not induce enhanced seeding ability for α-Syn fibrilization (Figures 2A,B and Supplementary Figures 1A,B). Western blots showed that the α-Syn PFFs-induced hyperphosphorylation of α-Syn at S129 was aggravated by SiO_2_ NPs (Figures 2C,D). To figure out whether SiO_2_ NPs promote α-Syn phosphorylation in primary neurons derived from α-Syn A53T Tg mice. Immunofluorescence analysis was performed and we found that more phosphorylated α-Syn appeared within the SiO_2_ NPs-treated neurons (Figures 3A,B). We further verified this result in SH-SY5Y cells. SiO_2_ NPs potent to promote endogenous α-Syn phosphorylation and aggregation in a dose-dependent manner, while antioxidant N-acetylcysteine (NAC) inhibited the aggregation of α-Syn induced by the combination of SiO_2_ NPs and α-Syn (Figures 3C,D). What’s more, cells treated with 200 μg/mL SiO_2_ NPs exhibited more pα-Syn aggregation compared with the H_2_O_2_ group (Figures 3C,D). In conclusion, SiO_2_ NPs significantly promote α-synucleinopathy in HEK293-α-Syn cells and neurons, and inhibition of oxidative stress attenuates the aggregation of α-Syn.

### Silica Nanoparticles Induce Mitochondrial Dysfunction and Oxidative Stress

**FIGURE 4:**
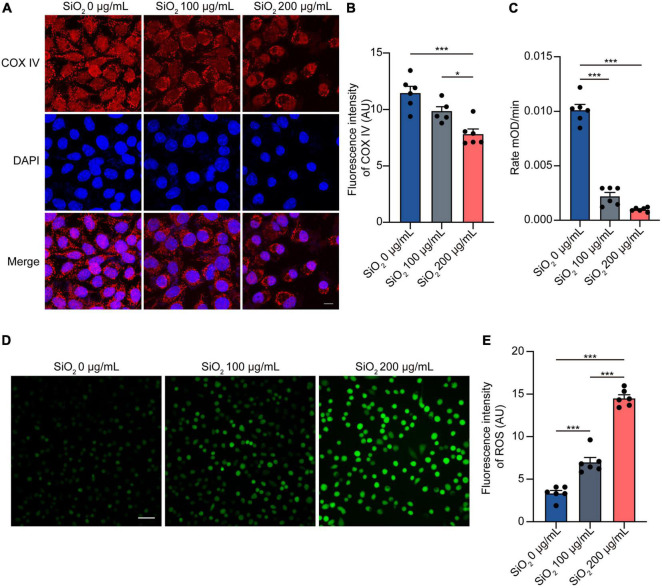
SiO_2_ NPs induce mitochondrial dysfunction and oxidative stress in SH-SY5Y cells. **(A,B)** Immunofluorescence staining of COX IV in SH-SY5Y cells treated with SiO_2_ NPs, scale bar = 10 μm (mean ± SEM; *n* = 6 per group; *P < 0.05, ***P < 0.001, one-way ANOVA). **(C)** Complex I enzyme activity of SH-SY5Y cells treated with SiO_2_ NPs (mean ± SEM; *n* = 6 per group; *P < 0.05, ***P < 0.001, one-way ANOVA). **(D,E)** ROS staining of SH-SY5Y cells treated with SiO_2_ NPs, scale bar = 50 μm (mean ± SEM; *n* = 6 per group; ***P < 0.001, one-way ANOVA).

Increasing evidence states that mitochondrial dysfunction is at the core of the pathogenesis of PD (Monzio Compagnoni et al., 2020). To investigate if SiO_2_ NPs assert their negative effects by impairing mitochondria, we treated SH-SY5Y cells with SiO_2_ NPs of different concentrations. Immunofluorescence analysis showed that the expression of mitochondrial biomarker COX IV significantly decreased after SiO_2_ NPs treatment (Figures 4A,B). Exposure to SiO_2_ NPs reduced the bioactivity of mitochondrial Complex I (Figure 4C). Oxidative stress usually is activated following mitochondrial dysfunction. Thus, we tested the effects of SiO_2_ NPs on ROS surging. More ROS-positive cells were observed in the SiO_2_ NPs-treated SH-SY5Y cells (Figures 4D,E). Collectively, SiO_2_ NPs induce mitochondrial dysfunction and oxidative stress in SH-SY5Y cells.

### Silica Nanoparticles Inhibit Autophagy and Promote Apoptosis

**FIGURE 5:**
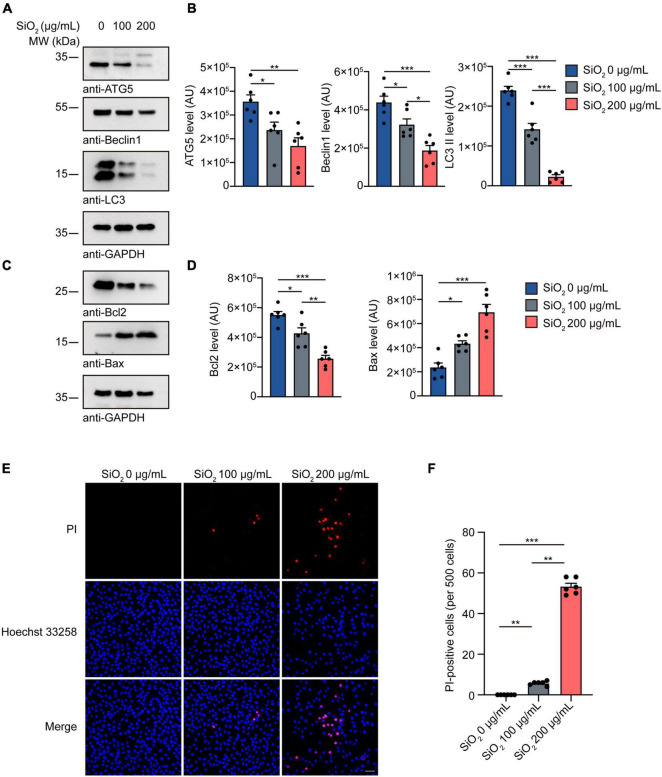
SiO_2_ NPs inhibit autophagy and promote apoptosis in SH-SY5Y cells. **(A,B)** Western blot of ATG5, Beclin1, and LC3 in SH-SY5Y cells treated with SiO_2_ NPs (mean ± SEM; *n* = 6 per group; *P < 0.05, **P < 0.01, ***P < 0.001, one-way ANOVA). **(C,D)** Western blot of Bcl2 and Bax in SH-SY5Y cells treated with SiO_2_ NPs (mean ± SEM; *n* = 6 per group; *P < 0.05, **P < 0.01, ***P < 0.001, one-way ANOVA). **(E,F)** PI/Hoechst 33258 staining of SH-SY5Y cells treated with SiO_2_ NPs, scale bar = 50 μm (mean ± SEM; *n* = 6 per group; **P < 0.01, ***P < 0.001, one-way ANOVA).

Autophagy impairment contributes to the pathogenesis of PD (Bellomo et al., 2020). To investigate the toxicity of SiO_2_ NPs on hindering the physiological autophagy process, we quantified the autophagy intensity in SH-SY5Y cells. Western blots showed that the expression of autophagy-related protein LC3 II, Beclin1, and ATG5 was significantly decreased in the SiO_2_ NPs-treated group (Figures 5A,B), suggesting an autophagy abnormality. And then we tested the cell apoptosis caused by SiO_2_ NPs using SH-SY5Y cells. The content of Bax increased in a dose-dependent manner after the adding of SiO_2_ NPs (Figures 5C,D). However, the expression of Bcl2 was significantly decreased. PI/Hoechst staining verified that SiO_2_ NPs induced apoptosis in SH-SY5Y cells (Figures 5E,F). In a word, SiO_2_ NPs induce pathological autophagy and cell apoptosis.

### Silica Nanoparticles Promote α-Synuclein Pathology and Degeneration of Dopaminergic Neurons in α-Synuclein A53T Tg Mice

**FIGURE 6:**
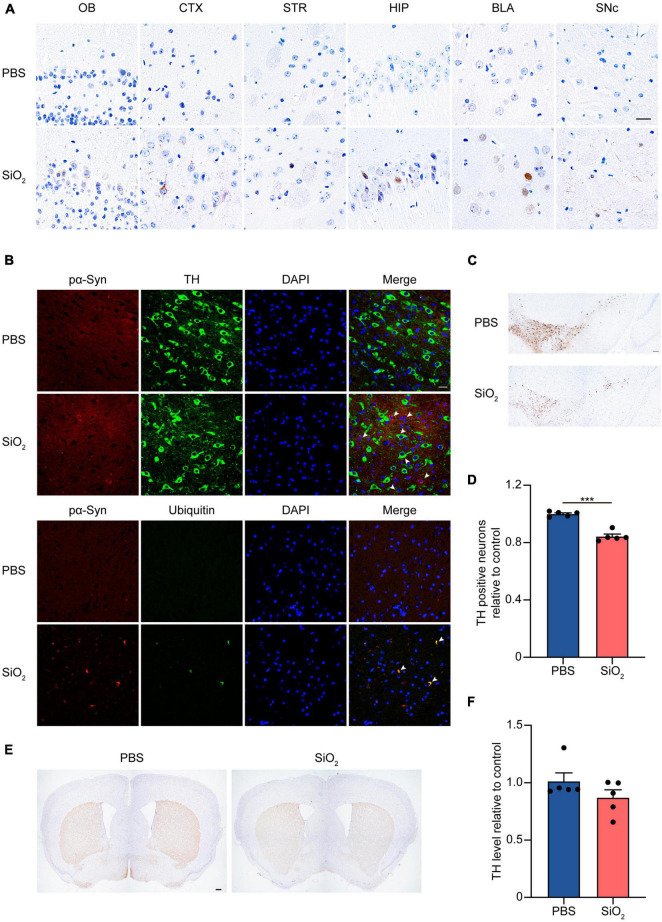
SiO_2_ NPs aggravate α-Syn pathology in α-Syn A53T Tg mice. **(A)** Immunohistochemistry of pα-Syn in brain sections, scale bar = 20 μm. **(B)** Immunofluorescence staining of TH, ubiquitin, and pα-Syn in brain sections, scale bar = 20 μm. **(C,D)** Immunohistochemistry showing the number of TH-positive dopaminergic neurons in the SNpc. Scale bar = 100 μm (mean ± SEM; *n* = 5 mice per group; ***P < 0.001, Student’s *t*-test). **(E,F)** Immunohistochemistry showing the density of TH-positive dopaminergic terminals in the striatum, scale bar = 200 μm (mean ± SEM; *n* = 5 mice per group).

To investigate the effects of SiO_2_ NPs on inducing the aggregation of α-Syn *in vivo*, we intranasally delivered the SiO_2_ NPs to 3-month-old α-Syn A53T Tg mice for 3 months. Immunohistochemistry showed that more pα-Syn was detected in PD-associated brain regions including the striatum and the SN of mice in the SiO_2_ NPs group compared with the PBS-treated mice (Figure 6A). Double immunofluorescence using brain sections with anti-pα-Syn and anti-TH antibodies illustrated abundant intracellular α-Syn deposits within the residual dopaminergic neurons in the SN (Figure 6B). Generally, Lewy bodies in the brains of PD patients are highly ubiquitinated. We performed double-labeling immunofluorescence of ubiquitin and pα-Syn using mouse brain slices. A large amount of α-Syn colocalized with ubiquitin in the brains of the SiO_2_ NPs-treated group but not while the control group manifested much less α-Syn ubiquitylation (Figure 5B). We further tested the effects of SiO_2_ NPs on nigrostriatal degeneration. Anti-TH immunohistochemistry showed that the number of DA neurons in the SNpc was significantly reduced after SiO_2_ NPs administration (Figures 6C,D). The striatal DA terminals also underwent a remarkable degeneration after SiO_2_ NPs treatment (Figures 6E,F). In conclusion, SiO_2_ NPs promote PD-like pathology including α-Syn aggregation and dopaminergic neuronal degeneration in α-Syn A53T Tg mice.

## Discussion

Since SiO_2_ NPs are currently extensively applied in industrial processing due to their ideal dispersibility, highly tunable stability, and biocompatibility (Vance et al., 2015), the biological safety of SiO_2_ NPs has been gradually attracting increasing concerns. Recently, a study conducted in MPTP mice model found that intragastric administration of SiO_2_ NPs of 150 nm for 5 days does not affect the striatal dopamine levels, indicating that oral administration is a relatively safe way for nanocarrier for PD drugs (Guzman-Ruiz et al., 2019). However, SiO_2_ NPs are also the most common component of mineral dust and particulate matter, causing numerous health issues in susceptible cohorts, such as workers in industrial fields (You et al., 2018). SiO_2_ NPs were reported for a capability of invading brains through the nasal mucosa (Wu et al., 2011). After inhalation, SiO_2_ NPs can translocated to the brain via the olfactory nerve (You et al., 2018). Once in the brain, they may be toxic for neurons. SiO_2_ NPs could be intake by SH-SY5Y cells and primary cultured hippocampal cells (Ducray et al., 2017) and promote the releasing of inflammatory chemicals by activating macrophages and microglia (Du et al., 2019; Inoue et al., 2021). Intranasal exposure of SiO_2_ NPs induces dysfunction of the antioxidant system and upregulates the levels of TNF-α, IL-1β, and MCP-1 in rat brains (Parveen et al., 2017). Olfactory dysfunction is considered one of the earliest symptoms of PD and pathological evidence implies that the olfactory nucleus is the very first region deteriorated by α-synucleinopathy (Braak et al., 2003). These findings suggest that respiratory tract exposure of SiO_2_ NPs could be a risk factor for PD. Therefore, whether SiO_2_ NPs participate in the onset and progression of PD and the underlying mechanisms are still need to be elucidated. Here we showed that SiO_2_ NPs treatment promotes the aggregation of α-Syn in HEK293-α-Syn cells. After intranasal instillation with SiO_2_ NPs for 3 months, α-Syn A53T Tg mice exhibit more severe α-synucleinopathy compared with the control group. In conclusion, our results indicate that SiO_2_ NPs aggravate the development of PD-like pathology both *in vitro* and *in vivo*.

Mitochondria dysfunction contributes to the aggregation of α-Syn and the degeneration of dopaminergic neurons during the typical course development of PD (Nicoletti et al., 2021). And interestingly, mitochondrial dysfunction occurs at the very early phase of neurodegeneration (Yong-Kee et al., 2012). Energy metabolism disorder in PD might affect the microtubule depolymerization, protein oxidation, and finally promote the α-Syn oligomerization (Esteves et al., 2009). Our results showed that SiO_2_ NPs treatment significantly reduced the number of mitochondria and the activity of Complex I, a key component in the mitochondrial electron transport chain. All these facts indicated a remarkable neurotoxicity of SiO_2_ NPs. Besides, we also observed a significant increase in ROS, suggesting enhanced oxidative stress in SiO_2_ NPs-treated cells. Thus, SiO_2_ NPs cause mitochondrial dysfunction and oxidative stress, which might be associated with α-Syn aggregation.

Pathological autophagy is one of the critical features of PD. Autopsy of PD patients illustrated abnormal autophagy-related structures in neurons in the SNpc (Anglade et al., 1997). Oxidative stress and mitochondrial dysfunction promote pathological autophagy (De Gaetano et al., 2021). Under physiological conditions, α-Syn monomers are degraded by the ubiquitin-proteasome system. However, for PD cases, aggregated α-Syn are mainly degraded via another approach, the autophagy-lysosomal pathway (Pantazopoulou et al., 2021). Autophagy dysfunction affects the turnover of α-Syn and promotes its aggregation. We found that SiO_2_ NPs decreased expression of Beclin1, ATG5, and LC3, suggesting that pathological autophagy could be triggered by SiO_2_ NPs. Several previous studies found that some nanoparticles enhanced autophagy in cells and animals (Murugadoss et al., 2017). This discrepancy may be caused by distinct kinds of nanoparticles. After exposure to nanoparticles, autophagy was activated to remove these exogenic materials. Nanoparticles were found in the endoplasmic reticulum in SH-SY5Y cells treated with SiO_2_ NPs (Ducray et al., 2017). In a word, since SiO_2_ NPs might occupy the majority of clearance capacity of autophagy system, this SiO_2_ NPs-induced overload would significantly reduce the clearance efficacy to α-Syn aggregates.

One most important feature of PD is the cell-to-cell trans-synaptic spreading of α-Syn. Many studies have shown that α-Syn pathology can spread bidirectionally along the nerve, further inducing various symptoms (Van Den Berge et al., 2019; Ferreira et al., 2021a; Jan et al., 2021). The spreading of misfolded α-Syn is affected by multiple factors, including cellular environment (Jan et al., 2021), mitochondrial dysfunction (Nicoletti et al., 2021), autophagy defects (Cheng et al., 2020), neuroinflammation (Tiwari and Pal, 2017), and α-Syn binding proteins (Ferreira et al., 2021b). Supporting this, SiO_2_ NPs were proved in our study to induce mitochondrial dysfunction and autophagy defects, further promoting α-Syn aggregation and propagation. In addition, intracellular post-translational modifications (PTMs) and some intracellular proteins have been shown involving in the formation of different α-Syn strains, which might be responsible for the clinical heterogeneity of PD and related α-synucleinopathies (Ma et al., 2016; Jan et al., 2021). For example, α-Syn inclusions isolated from multiply system atrophy brains have different ultrastructural features from those of PD brains (Peng et al., 2018). What’s more, p25α, an oligodendroglial protein, can redirect α-Syn aggregation into a unique α-syn/p25α strain, which enhanced neurodegenerative properties *in vivo* (Ferreira et al., 2021b). Our research focused on the changes of SiO_2_ NPs to the cellular environment. Whether SiO_2_ NPs have effects on the conformation of α-Syn fibrils needs further investigation.

## Conclusion

A lot of potential mechanisms participate in the pathogenesis of PD, such as the misfolded α-Syn aggregation, mitochondrial dysfunction, oxidative stress, and autophagy. Here we found that SiO_2_ NPs promote the aggregation of α-Syn, mitochondrial dysfunction, oxidative stress, autophagy impairment. Furthermore, SiO_2_ NPs exacerbate PD-like pathology in the α-Syn A53T Tg mice. These observations may provide new evidence for investigating the potential risk of SiO_2_ NPs exposure on triggering PD.

## Data Availability Statement

The original contributions presented in the study are included in the article/Supplementary Material, further inquiries can be directed to the corresponding author.

## Ethics Statement

The animal study was reviewed and approved by the Laboratory Animal Welfare Ethical Committee (IACUC) of Renmin Hospital of Wuhan University.

## Author Contributions

ZZ conceived the project and designed the experiments. XY and YY performed most of the experiments. DX participated in manuscript writing. LM, MH, and CL participated in data analysis. All authors have read and approved the final manuscript.

## Conflict of Interest

The authors declare that the research was conducted in the absence of any commercial or financial relationships that could be construed as a potential conflict of interest.

## Publisher’s Note

All claims expressed in this article are solely those of the authors and do not necessarily represent those of their affiliated organizations, or those of the publisher, the editors and the reviewers. Any product that may be evaluated in this article, or claim that may be made by its manufacturer, is not guaranteed or endorsed by the publisher.

## Supplementary Material

Supplementary Figure 1 Effects of different SiO_2_ on HEK293-α-Syn cells. **(A,B)** Fluorescence analysis showing α-Syn aggregates in HEK293-α-Syn cells treated with different SiO_2_ and α-Syn PFFs. Scale bar = 20 μm (mean ± SEM; *n* = 6 per group; ***P* < 0.01, ****P* < 0.001, one-way ANOVA).
