# Supplementary figures and images for: Silica Nanoparticles Promote α-Synuclein Aggregation and Parkinson’s Disease Pathology
Source: Front Neurosci. 2022 Jan 13;15:807988. doi: 10.3389/fnins.2021.807988 (PMC8792744; doi:10.3389/fnins.2021.807988)

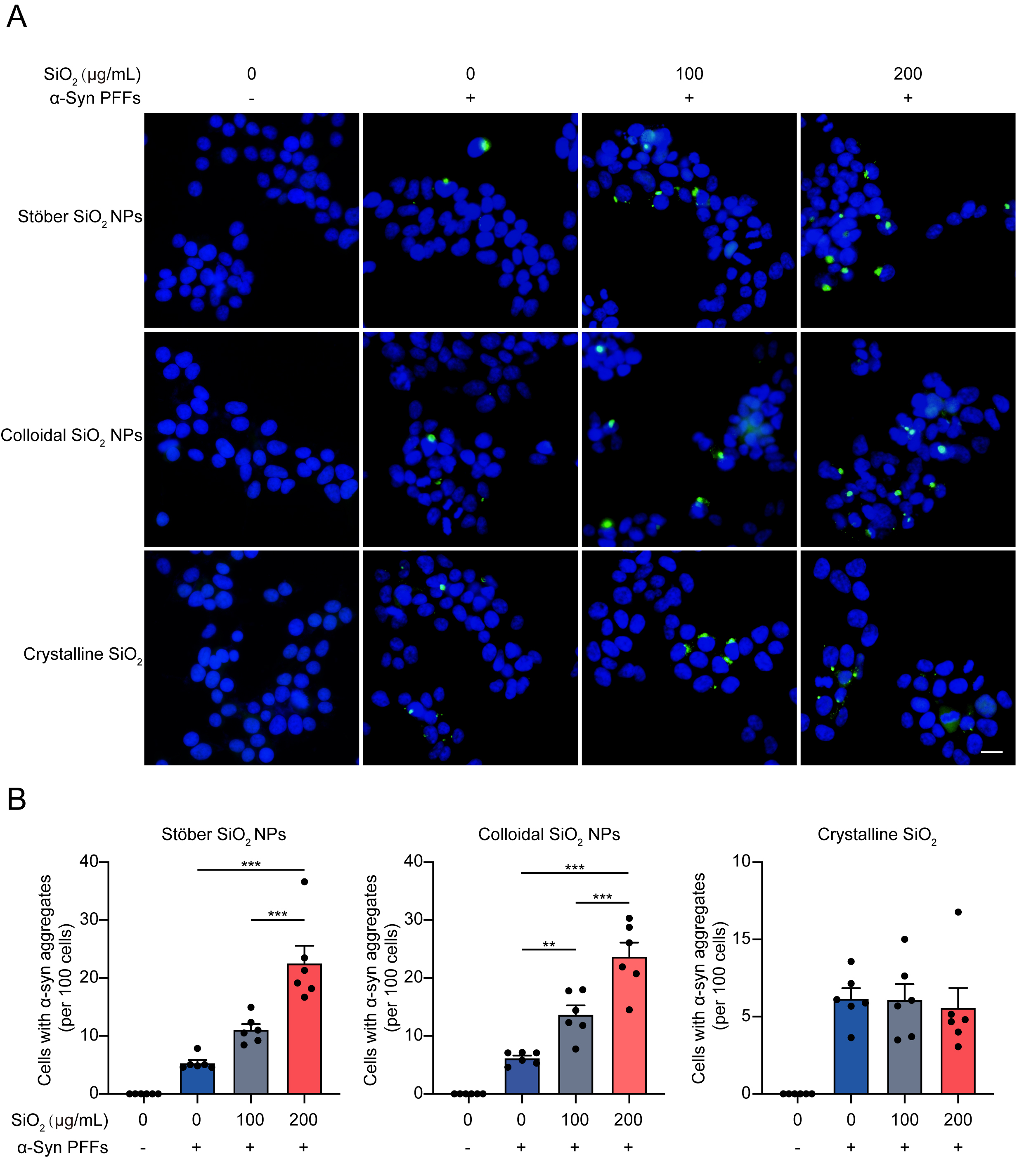

Supplement: Supplementary Figure 1 — Effects of different SiO2 on HEK293-α-Syn cells. (A,B) Fluorescence analysis showing α-Syn aggregates in HEK293-α-Syn cells treated with different SiO2 and α-Syn PFFs. Scale bar = 20 μm (mean ± SEM; n = 6 per group; **P < 0.01, ***P < 0.001, one-way ANOVA). [file Image_1.JPEG]
